# An etude for post-pandemic practice: The impact of the COVID-19 pandemic on practice methods and instrumental technique

**DOI:** 10.3389/fpsyg.2022.846953

**Published:** 2022-12-21

**Authors:** Ellen Fallowfield, Patrick Gomez

**Affiliations:** ^1^Department of Music, Hochschule der Künste Bern, Berner Fachhochschule, Bern, Switzerland; ^2^Department of Music, Hochschule für Musik Basel, Fachhochschule Nordwestschweiz, Basel, Switzerland; ^3^Center for Primary Care and Public Health (Unisanté), Department of Occupational and Environmental Health, University of Lausanne, Lausanne, Switzerland

**Keywords:** musical instrument practice, instrumental technique, COVID-19, music education, digital tools

## Abstract

This paper considers how the pandemic-related concert-free time affected musicians’ practice, specifically in relation to technique. A semi-structured interview was carried out on 22 musicians based in Switzerland (11 males, 11 females; 7 students, 15 non-students; 11 with school-aged children, 11 without school-aged children; 16 with teaching duties and 6 non-teachers). The amount of practice during the pandemic-related concert-free time was reported as different to usual by 91% and usual for only 9% of participants (*p* = 0.002). Forty-one percent of participants reported reduced, and 41% “fluctuating” amounts of practice. The proportion of practice time spent on technique was reported by 55% of participants to have increased and by only 9% to have decreased (*p* = 0.019). Of those who reported an increase in technique practice, 75% agreed this had a positive impact on technique, and only 8% disagreed (*p* = 0.037). Moreover, 58% considered this work to have changed their current and future practice. Participants were statistically more likely to report “never” watching online tutorials than “often” (*p* = 0.014), but, of those that did watch such material, 75% agreed that it had a positive impact upon their practice. Most participants created digital content during this period; only 5% produced no such material. An increased use of digital tools was reported by 55% of participants, 92% of whom described this as having a positive effect upon practice and only 8% were unsure (*p* = 0.022). However, in the unstructured discussion, the use of digital tools appears to be associated with mixed outcomes. Men reported significantly more frequent use of digital tools (91% vs. 45% describing this use as often, *p* = 0.038) and spent a larger proportion of time on technique relative to their pre-pandemic habits than women (*p* = 0.065); moreover, a trend indicated that more women than men created digital content in the form of tutorials (*p* = 0.095). The exceptional situation musicians experienced during the pandemic, which introduced new aspects to musical instrument practice, and accelerated changes already underway, could lead to future work that improves practice under “normal” conditions, and exposes discrepancies between certain demographic groups.

## Introduction

Studies of musicians’ wellness, practicing routines, and higher education provision during the COVID-19 pandemic-related lockdowns have pointed to a complex situation that, despite obvious disadvantages, has also been described by some musicians as a creative period. Indeed, evidence of musicians’ creativity during this period can be seen in a surge of online material, including concert streams, and self-made videos on technique and practice tips.

The impact upon musicians of the COVID-19 pandemic, specifically the lockdown periods during which live concerts and in-person music teaching were prohibited in many countries, has been evaluated in the literature from various perspectives. Despite prevailing negative themes such as musicians’ loss of income and reduced wellness, reactions to other effects of the pandemic lockdown, such as the use of technology in music teaching, seem to be more complex, with musicians able to identify both positive and negative aspects. Performing arts professionals reported reduced work and income, reduced wellbeing, and specific challenges, such as those associated with caring responsibilities (Spiro et al., 2021). Musicians reported practicing less, but also described using the lockdown to work creatively, e.g., learn new skills and make social connections (Spiro et al., 2021). A comparison of the pandemic’s impact upon the professional lives and wellbeing of mid-career freelancers vs. an older, more experienced cohort showed that, while musicians from both groups described career-related concern and financial stress, the mid-career respondents reported more distress, specifically citing less practice time, less engagement with collaborative music-making, and getting less pleasure from the musical activities they were undertaking (Cohen and Ginsborg, 2021).

A significant body of research focuses on the digitalization of teaching at conservatoire-level during the pandemic, detailing the technical demands, and the resulting impact on teachers and students, including influence on time spent practicing. An investigation into the effect of the shift to online classes and asynchronous learning formats at the Music University of Freiburg showed that some students practiced less during the pandemic compared to a similar group of students pre-pandemic. However, the type of practice was identified as being more self-motivated and more effective at accomplishing goals than pre-pandemic practice, and thus, the experience was not perceived to be wholly negative (Nusseck and Spahn, 2021). The perspective of teachers at French and Swiss conservatoires was investigated specifically in relation to the move to digital teaching. Teachers were able to point to advantages and disadvantages of digital teaching, and described a long-term impact in their pedagogical practice. While the majority of interviewees reported offering some online teaching that exactly replicated what they would do in a classroom, the making and sharing of recordings was important too: the majority had commented on video and audio recordings made by students, and just over half had made video recordings of themselves. The teachers described this situation as having a positive influence upon their pedagogical approach, but also described significant disadvantages, especially not being able to play alongside students (Güsewell and Terrien, 2021). Another study reported fluctuating motivational levels in a group of 12 athletes and musicians, and highlighted various positive and negative issues in these students’ relationships with their coaches and teachers during the COVID-19 pandemic (Philippe et al., 2020). A survey at an Italian conservatoire studied changes in the educational program of 20 music students (Schiavio et al., 2021). Negative effects related to technology, such as technical issues, were reported in parallel with positive experiences such having more time to learn about new teaching technology, and eventually enjoying working with these formats. Moreover, the study participants used technology to actively seek connections with their fellow students during the period when in-person group classes did not take place. This points to the high value placed upon social life during studies, but also perhaps to the perceived importance of peer-peer learning that has been shown to exist amongst conservatoire students (Nielsen et al., 2018). Several of the above discussions reflect the pre-pandemic findings and recommendations made by Jørgensen (2015) in a report on a scheme whereby teacher-led pedagogy projects that sought unconventional ways to teach musical instrument practice were trailed at the Norwegian Academy of Music, and evaluated by students and staff. The post-study report describes a willingness to experiment on the part of both students and professors. Issues that were flagged included limitations in existing digital tools for storing and organizing recordings (Jørgensen, 2015). Making and commenting on video recordings of one’s own practice has been suggested to be an effective evaluation strategy in musical instrument practice, and a route to independent learning (Jørgensen, 2004), and audio recordings have been used to guide music students’ critical reflection of their own practice and promote self-regulated learning (McPherson et al., 2019). Practice strategies that include making and listening back to recordings, and using a metronome, have been associated with a high level of expertise in a study comparing young musicians of varying expertise (Hallam et al., 2012). A British study that surveyed a cohort of 338 amateur, student, and professional musicians reported that the drivers to new technology that support musical instrument performance are significantly higher than entry barriers to using such technology. Most musicians used tuning and metronome applications on mobiles rather than bespoke devices, and recorded themselves approximately once a week, although they did not always listen to their own recordings. Moreover, students listened to recordings of others more often than professionals did (Waddell and Williamon, 2019).

The connection between creativity and the use of digital resources, such as recording devices, has been investigated in several studies. Creativity has been suggested to be an important aspect of musical instrument practice, and the use of video “practice diaries” to self-evaluate creativity may improve problem-solving and increase agency in the practicing process (Wise et al., 2017). The creative use of technology to collaborate with other musicians, evidenced in a group of musicians from various genres, may have been an important factor in building resilience during the pandemic (Fram et al., 2021). The evaluation of digital resources used during the pandemic for music teaching, music making, and communication includes proposed qualities for improving connection and relationship-building in online teaching (de Bruin, 2021), reports of initial artistically positive experiences associated with increased creative time to practice and compose versus long-term fears about the lack of sustainability of digital music making in some cases, especially in an ensemble (Verhulst, 2021), and the creation and analysis of online platforms that generate music by connecting musicians, e.g., composers and performers (Stollery and Black, 2021).

In summary, existing research into musicians’ work and lifestyle during the COVID-19 pandemic generally reports reduced wellness, and reduced practice time, which, in some cases, was nonetheless described as being creative, and more efficient than usual practice. Reduced social mixing and inability to play together with colleagues were often reported as significant negatives; proposals to compensate for this *via* digital resources, such as making and sharing recordings, had mixed success. During the COVID-19 pandemic-related concert-free time, there was a surge in online streams, including pre-recorded and archival material, but also many live performances, from solo concerts in musicians’ living rooms to orchestras live-streamed from empty concert halls (see, e.g., Barone, 2020; Macdonald, 2020; Paar, 2020), and asynchronized recordings of individual, distanced musicians compiled together to create ensemble works (e.g., this contribution from the Rotterdam Philharmonic Orchestra^1^). This output extended beyond concerts to discussions and demonstrations, especially with technical tips (e.g., by cellist Ferrándes, 2020). Indeed, musicians have discussed using the lockdown, during which there was little or no requirement to practice ensemble repertoire, to work on technique (Gersten, 2020). This is the environment that this paper aims to explore. The study presented here has a different focus to existing studies that have investigated wellness, practice time, and music pedagogy; instead, technique-based practice and use of digital content and tools to aid practice during the COVID-19 pandemic-related concert-free time are considered. A short survey was undertaken to investigate how frequently musicians practiced during the concert-free time. Participants were asked what proportion of practice time was spent on technique, whether they consumed or created digital content related to technique, and how they used digital tools during this period.

## Aims

This study aims to investigate how the COVID-19 pandemic influenced aspects of musical instrument/vocal practice. The four main areas of interest are the following behaviors and activities during the concert-free time:

- The **amount of practice** that musicians were undertaking.
- The type of practice that musicians were undertaking, especially the **proportion of time spent practicing technique.**
- The **consumption and creation of audio/video content** related to technique.
- The **use of digital tools** in the practice room.

The goal was to compare this with pre-pandemic working habits, and determine whether any changes made to practice had a positive and/or lasting effect on practicing methods, and could thus be incorporated into future studies and practicing programs.

## Materials and methods

A semi-structured interview was developed and carried out between September and November 2021 in-person or on the telephone with 22 musicians living in Switzerland. A copy of the interview script can be found in the Supplementary material of this paper.

The questionnaire script was followed strictly during each interview, and consisted of a combination of fixed, targeted questions followed by open questions to allow more nuanced discussion. The interviewees were given time to comment freely at the end of each question. All musicians were living in Switzerland during the pandemic, so they experienced the same national measures restricting concert activities, in summary: a first lockdown prohibiting concerts and rehearsals from 16 March to 6 June 2020, a period with no restrictions, limitations to 15 performer/audience members in November/December 2020, a second lockdown from 22 December 2020 to 19 April 2021, limitations to audiences of up to 50 people from 19 April 2021, and lifting of all restrictions on 26 June 2021.^2^ The musicians also had available to them the same structure of financial compensation offered by the Swiss government.

The following definitions were given to the interviewees before the interview. “Concert-free time” was defined as the periods of complete lockdown or periods when no in-person concerts could take place (but, for example, concert streams with ensembles were possible). “Usual” practice was defined as that undertaken during a typical working period pre-pandemic. Participants were recruited in the following way: an e-mail invitation to participate that was sent to local ensembles and professional music associations. Positive respondents were asked whether they met the following criteria: residency in Switzerland during the pandemic, and a regular professional performance schedule (participants were allowed to decide for themselves whether their performance schedule was “regular,” if they questioned this further, they were asked to consider whether a significant amount of their average monthly income came from performance activities). From 23 responses, one was discarded due to residency outside of Switzerland. The remaining 22 respondents were interviewed. In addition to noting instrument type and student/professional status, a short demographic survey also recorded whether the participant taught, and if they have school-aged children. This demographic data is summarized in the Supplementary material Table S1 to allow the reader to connect the quotes in “Demographic trends” with the demographic data. Participants have been assigned the codes P1 to P22.

## Results

The results from the interviews are given below. “Main survey results” reports the responses to the semi-structured interview, “Demographic trends” describes some tendencies according to the participants’ demographics and “Additional comments” presents selected comments from the respondents’ free feedback that they were allowed to give at the end of each question.

We performed all statistical analyses using STATA version 16.1 for Windows (Stata Statistical Software; StataCorp LP, College Station, TX, United States). For the results reported in “Main survey results”, we used multinomial logistic regression to estimate the odds of each answer relative to the other answers (i.e., relative risks). For the results reported in “Demographic trends,” we used Fisher’s exact and ordered logistic regression (for ordinal variables) and logistic regression (for binary variables) to evaluate the differences in the distribution of the answers to the different questions as a function of student/professional status (students vs. non-students), having school-aged children (children vs. no children), teaching activity (teach vs. do not teach), and gender (female vs. male). For these latter analyses, we only considered the questions that were answered by all 22 participants.

### Main survey results

The survey results are presented in the four main sections of the survey, respectively: Amount of practice, Type of practice, Online content, and Digital tools. For some questions, participants who responded in a certain way were asked to answer a follow-up “sub-question.” This is delineated in the text below. The descriptive statistics are summarized in Table 1. The results of the statistical tests are presented in the Supplementary material.

**Table 1:** Practice habits during the pandemic-related concert-free time compared with usual habits pre-pandemic.

Percent (#participants)						
Amount of practice compared to usual						
More	Less	The same	Fluctuating			
9% (2)	41% (9)	9% (2)	41% (9)			
Proportion of practice time spent on technique compared to usual						
Larger	Smaller	The same				
55% (12)	9% (2)	36% (8)				
				Subquestions: if answer above was “Larger,” agreement with the following statements		
				This has had a positive impact on my technique		
				Agree	Disagree	Not sure
				75% (9)	8% (1)	17% (2)
				This has changed how I practice now, and will practice in the future		
				Agree	Disagree	Not sure
				58% (7)	25% (3)	17% (2)
Frequency of watching/reading online content related to technique						
Often	Sometimes	Never				
9% (2)	32% (7)	59% (13)				
Amount of content compared to usual habits						
More	Less	The same				
36% (8)	0	64% (14)				
Breakdown: those who answered “Often” above						
100% (2)	0	0				
Breakdown: those who answered “Sometimes” above						
86% (6)	14% (1)	0				
Breakdown: those who answered “Never” above						
0	0	100% (13)				
				Subquestions: if answer above was “More,” agreement with the following statements		
				Watching/reading this content has had a positive impact upon how I practice		
				Agree	Disagree	Not sure
				75% (6)	0	25% (2)
				The majority of this content was produced by my peers		
				Agree	Disagree	Not sure
				25% (2)	75% (6)	0
Participation in streamed/pre-recorded online activities						
Concerts only	Tutorials only	Both	None			
50% (11)	18% (4)	27% (6)	5% (1)			
Frequency of use of digital tools (metronome, tuner, recording device) during practice						
Often	Sometimes	Never				
68% (15)	27% (6)	5% (1)				
Use compared to usual habits						
More	Less	The same				
54% (12)	5% (1)	41% (9)				
Breakdown: those who answered “Often” above						
67% (10)	0	33% (5)				
Breakdown: those who answered “Sometimes” above						
33% (2)	0	67% (4)				
Breakdown: those who answered “Never” above						
0	100% (1)	0				
				Subquestion: if answer above was “More,” agreement with the following statement		
				Using these digital tools has had a positive impact upon how I practice		
				Agree	Disagree	Not sure
				92% (11)	0	8% (1)

#### Amount of practice

The participants were asked how the amount of time they spent practicing during the concert-free time compared with their usual, pre-pandemic routine. The amount of practice during the pandemic-related concert-free time was reported as different to usual by 91% of participants and usual for only 9% of participants (significantly different odds, *p* = 0.002, see Table S2 in the Supplementary material). Specifically, 41% of participants reported practicing less, and the same percentage said they would describe their practice as “fluctuating,” i.e., they agreed with the statement: “*there were periods when I practiced a lot more than usual, and periods when I practiced a lot less than usua*l.” Nine percent of participants reported practicing more than usual.

#### Type of practice

Technique practice was defined to be “*etudes, studies, exercises, scales, repertoire excerpts with a technical goal, general technical work on sound/intonation*, etc.” When asked to consider the proportion of practice time spent on technique, 55% of respondents reported this to be larger than usual, 36% “the same” and 9% “smaller.” The odds of answering “larger than usual” were significantly higher than the odds of answering “smaller than usual” (*p* = 0.019, see Table S3 in the Supplementary material).

Sub-question: Those who reported a larger proportion of practice time spent on technique were asked whether they agreed, disagreed or were not sure whether the following statements were true:

‘This has had a positive impact on my technique’

‘This has changed how I practice now, and will practice in the future’

Seventy-five percent (of those who reported a larger proportion of practice time spent on technique) agreed with the former statement and 58% with the latter statement. The odds of agreeing with the former statement were significantly higher than the odds of disagreeing with it (*p* = 0.037, see Table S4 in the Supplementary material). There were no significant differences in the odds of the answers to the latter statement (see Table S5 in the Supplementary material).

#### Online content

The participants were asked if they had watched or read online content relating to technique. This was defined within the questionnaire as including masterclasses, tutorials, podcasts, blogs, and articles, but excluding concerts. Fifty-nine percent of respondents reported never watching or reading such content, and all this group said that this was in line with their usual habits. Thirty-two percent of respondents reported watching or reading such content “sometimes,” of which 14% reported this to be “the same” and the remaining 86% “more” than their usual habits. Nine percent of respondents reported watching or reading such content “often,” all of whom described this as “more” content than their usual habits. The odds of answering “never” were significantly higher than the odds of answering “often” (*p* = 0.014, see Table S6 in the Supplementary material). There were no significant differences in the odds of whether the use of the online content was more, the same, or less than usual (see Table S7 in the Supplementary material).

Sub-question: Respondents who reported watching/reading more online content than usual were asked if they agreed, disagreed or were unsure if they agreed with two follow-up statements:

The majority of this content was produced by my peers (musicians around the same age as me, undertaking similar activities to me).

Watching/reading this content has had a positive impact upon how I practice.

As seen in Table 1, 75% of respondents who had reported watching/reading more online content disagreed that the majority of this content was produced by peers, 15% agreed with this statement, and no respondents were “unsure.” Seventy-five percent agreed that this content had a positive impact upon how they practiced, 15% said they were “unsure” whether they agreed with the statement, and no respondents answered “disagree.” No relative risk reached statistical significance for these two questions (see Tables S8 and S9 in the Supplementary material).

The interviewees were asked about the type of content they had created themselves: 27% reported having taken part in streamed or pre-recorded concerts and created video tutorials, masterclasses, or blogs. Fifty percent of respondents had taken part in concerts only, 18% in tutorials only, and only 5% had done neither. The odds of having created streamed or pre-recorded concerts were significantly higher than the odds of not having created such content (*p* = 0.016, see Table S10 in the Supplementary material). In contrast, the odds of having created or not video tutorials, masterclasses, or blogs did not significantly differ from each other (see Table S11 in the Supplementary material).

#### Digital tools

In response to whether the participants had used digital tools such as a recording device, metronome, or tuner, 68% described this use as “often,” with only 5% reporting “never,” and 27% “sometimes.” The odds of answering “often” were significantly higher than the odds of answering “never” (*p* = 0.009, see Table S12 in the Supplementary material). Fifty-four percent described this as being “more” than their usual habits (67% of whom had answered “often” above, and 33% “sometimes”), 5% reported “less” (answered “never” above) and the remaining 41% “the same” (33% of whom had answered “often” above, and 67% “sometimes”), as shown in Table 1. The odds of answering “more” were significantly higher than the odds of answering “less” (*p* = 0.017), and the odds of answering “the same” were significantly higher than the odds of answering “less” (*p* = 0.037, see Table S13 in the Supplementary material).

Sub-question: Those who reported more use of digital tools during the pandemic were asked whether they agreed with the statement *This has had a positive impact upon how I practice*. Ninety-two percent of those who had reported more use of digital tools agreed with this statement, 8% was not sure, and no one disagreed. The odds of agreeing with this statement were significantly higher than the odds of being unsure/disagreeing (*p* = 0.022, see Table S14 in the Supplementary material).

### Demographic trends

Despite the small sample size, some demographic trends may be indicated by the data. These are summarized in Table 2.

**Table 2:** Demographic breakdown: Practice habits during the pandemic-related concert-free time compared with usual habits pre-pandemic habits.

Percent (#participants)					
Subgroup	% of population (#participants)	More	Less	The same	Fluctuating
Amount of practice compared to usual					
Students	32% (7)	14% (1)	14% (1)	0	71% (5)
Non-students	68% (15)	7% (1)	53% (8)	13% (2)	27% (4)
Children	50% (11)	0	55% (6)	9% (1)	36% (4)
No children	50% (11)	18% (2)	27% (3)	9% (1)	45% (5)
Teach	73% (16)	6% (1)	50% (8)	13% (2)	31% (5)
Do not teach	27% (6)	17% (1)	17% (1)	0	66% (4)
Female	50% (11)	0	64% (7)	9% (1)	27% (3)
Male	50% (11)	18% (2)	18% (2)	9% (1)	55% (6)
**Subgroup**	**% of population (#participants)**	**Larger**	**Smaller**	**The same**	
Proportion of practice time spent on technique compared to usual					
Students	32% (7)	71% (5)	0	29% (2)	
Non-students	68% (15)	47% (7)	13% (2)	40% (6)	
Female	50% (11)	36% (4)	18% (2)	46% (5)	
Male	50% (11)	73% (8)	0	27% (3)	
**Subgroup**	**% of population (#participants)**	**Yes, created concerts**			
Participation in streamed/pre-recorded online activities—concerts					
Students	32% (7)	100% (7)			
Non-students	68% (15)	67% (10)			
**Subgroup**	**% of population (#participants)**	**Yes, created tutorials**			
Participation in streamed/pre-recorded online activities—tutorials/masterclasses					
Female	50% (11)	65% (7)			
Male	50% (11)	27% (3)			
Teach	73% (16)	56% (9)			
Do not teach	27% (6)	17% (1)			
**Subgroup**	**% of population (#participants)**	**Often**	**Sometimes**	**Never**	
Frequency of use of digital tools (metronome/tuner/recording device) during practice					
Students	32% (7)	100% (7)	0	0	
Non-students	68% (15)	53% (8)	40% (6)	7% (1)	
Female	50% (11)	45% (5)	45% (5)	9% (1)	
Male	50% (11)	91% (10)	9% (1)	0	

Participants with children, and those who teach reported practicing less than their counterparts during the concert-free time: less practice was reported by 55% of participants with children (vs. 27% of participants without children), and 50% of those who teach (vs. 17% of those who do not teach). Students reported practicing more than non-students: 14% (vs. 7% of non-students) reported practicing more, and 71% (vs. 27% of non-students) practiced in fluctuating amounts. When organized according to gender, the results show men practicing more than women: 18% of men (vs. 0 women) reported practicing more, 18% less (vs. 64% of women), and 55% a fluctuating amount (vs. 27% of women). No statistical test reached significance for any of these effects (see Table S15 in the Supplementary material).

In terms of proportion of practice time spent on technique, gender also appears to divide the participants, with 73% of men spending more time on technique than usual (vs. 36% of women), and no men (vs. 18% of women) spending a smaller proportion of practice time on technique. Students reported spending a larger proportion of practice time on technique than non-students (71% vs. 47%). None reported spending a smaller proportion of time on technique (vs. 13% of non-students) and 29% reported the same amount (vs. 40% of non-students). No statistical test reached significance for any of these effects (the gender effect approached significance, *p* = 0.065; see Tables S16 and S16.1–4).

For the questions regarding frequency of watching online content related to technique, and how this content compared to usual pre-pandemic habits, no discernible trend can be seen, and accordingly the statistical tests were far from significance (see Tables S17, S17.1–4, S18, and S18.1–4).

Most participants were active in creating their own online content in the form of concerts or tutorials; only 5% created no such content. Regarding the demographic breakdown, no statistical test reached significance for any of these effects; however, students appeared to be more likely to have created concert streams than non-students (100% vs. 67%, see Table S19.1 in the Supplementary material). Moreover, teachers were more likely to have created online tutorials than non-teachers (56% vs. 17%, see Table S20.3 in the Supplementary material), and there appears to be a trend whereby women were more likely to have created online tutorials than men (65% vs. 27%, *p* = 0.095, see Table S20.4 in the Supplementary material).

A frequent use of digital tools during practice was seen in the student population (100% reported using such tools often vs. 53% of non-students). Similarly, a larger proportion of men reported regular use of digital tools than women: 91% of men reported “often” vs. 45% of women, 9% of men vs. 45% women reported “sometimes” and 0 men vs. 9% of women reported never. The odds of using digital tools more often were significantly higher for men than for women (*p* = 0.038, see Table S21.4 in the Supplementary material). Regarding the question of how this use of digital tools compared to pre-pandemic practice, no statistical test reached significance (see Tables S22 and S22.1–4).

### Additional comments

After each question in the survey, respondents were given the opportunity to expand their answers with additional comments that they considered relevant. Below, selected comments that represent this feedback have been grouped according to recurring themes. Participants have been labelled with codes P1 to P22, and references to instrument and demographic information can be seen in the Supplementary material Table S1.

#### Amount of practice

The following quotes describe how time spent practicing was reduced by energy and time constraints caused by teaching and family commitments:

*P16*: I felt I was locked in a room with a computer, camera, microphones [because of online teaching] and afterwards there was absolutely no energy or interest to practice for myself.

*P4*: The experience of practice was different for those who have children and those who don’t have children…we were spending a lot of time with our kids and maybe not practicing as much.

Descriptions of fluctuating practice, as seen below, were typical:

*P17*: There were periods where I didn’t practice at all, and periods where I practiced regularly, about 3 hours a day.

Some comments showed participants to be relaxed and positive about the idea of practicing less than usual:

*P15*: Before the pandemic I had to practice almost every day to feel like I was doing well…but, actually, [I learnt that] taking care of my instrument [voice] also means resting sometimes.

#### Type of practice

Some respondents related an increased proportion of practice time spent on technique to not needing to prepare concert repertoire, or, as seen in the last example, practicing repertoire in a more technical way rather than preparing it for a performance, and spoke positively about this time:

*P22*: I had no specific things that I had to rush-practice for, so I had time to [work on technique].

*P19*: I had wanted to improve some [technical] things for a long time, and…finally I had the time to do it.

*P3*: Even when I did not practice technical stuff, I would practice pieces in quite a technical way.

The following comment describes a more ambiguous situation in which technical practice was viewed as having helped the participant but, nonetheless, overall technical level was perceived to have dropped:

*P4*: [Because I was practicing so little] I felt like my level was less good, even though I was doing technique exercises… that probably kept me on a better level than I would have been otherwise.

In contrast to the technical work described above, other respondents used the time to work on repertoire, either learning new pieces, or playing old pieces for their own enjoyment. For example:

*P8*: I practiced perhaps less, but …I had time that I could spend slowly on the pieces that…I had always wanted to play.

Further evidence of productivity and creativity during this time can be seen in several participants learning new instruments, practicing different types of music than usual, or getting involved in other aspects of musical life. For example:

*P6*: I started playing Arabic instruments during the pandemic.

*P14*: I was practicing other styles of music, pop and rock,…which was a type of technical work.

*P15*: I did a lot of interviews for podcasts via Zoom…it was not about music making but about the business of music making and kind of activism as well.

#### Online content

The period when streamed concerts were allowed was reported by several participants to have boosted the amount of practice undertaken:

*P3*: In the first lockdown when there were no streams I definitely practiced less. In the second lockdown, when the rules were a bit different, for example, when orchestras could do streams without an audience, then I practiced more.

The following quotes describe positive and more ambiguous feelings towards the results and the process of making video recordings for students:

*P18*: Every week I made a video for my students about Peter and the Wolf…it worked really well…

*P16*: Space is incredibly important for me, for teaching and for playing together, and having no space [because we were doing everything online or through recordings] really messed me up…In the end the effect was positive because you can learn from every new situation, but I’m happy that I don’t **have** to do it now

Respondents who reported producing online tutorials and masterclasses spoke positively about this process both when they listened back to the results, and when they did not. For example:

*P22*: I did a practice blog once a week every week during the pandemic…you practice way more intensely [with a live camera recording]…occasionally I would go back and [watch] the video…just to compare the progress for myself…but I didn’t regularly do that…maybe once or twice a month.

*P19*: I was getting paid [to make masterclasses in video form]…mostly [about] technical things. [I had often been told by teachers to record myself but] I didn’t take the time to actually do that. When I had to send some videos [for these masterclasses] then of course I was checking the videos first, and then I realised some things [about my playing], so it helped.

#### Digital tools

Recording devices featured regularly in the discussion about which types or digital tool participants used most during lockdown. The corresponding feedback shows a complex relationship, for example:

*P6*: [I made recordings of myself and watched them in slow motion], it’s better than a mirror because it’s delayed.

*P7*: Recording can be a fantastic tool. It makes you hear and see yourself from the outside, which gives you a kind of clarity…but it can make you afraid of playing the next note. It can be too objective [especially with limited quality microphones]… In many ways it made me love playing a lot less…and for a period of time changed the way I played to a way that sounded better on a recording but not in real life.

One participant described the use of a metronome not only as a tool to keep time, but also to keep focus:

*P3*: I definitely practice more concentrated [with a metronome]…keeping me focused on what I’m doing.

Several participants described investing in new technology during the pandemic, including specific software programs:

*P13*: We used a [programme for practicing ensemble pieces where each person records their part with a metronome] as a tool to improve…a programme of really difficult music… it was an amazing discovery how effective it could be to practice with this kind of digital tool…we would never have thought to have used it…we will use it again [and have found another programme with even better sound quality].

## Discussion

There follows a discussion according to the four focal points of this study, a summary of the study’s limitations, and proposals for future work.

### Amount of practice

In this study, 91% of participants reported a change in amount of practice compared to their pre-pandemic habits. Most of the 22 participants described this change as practicing less or in fluctuating amounts (41% in both cases). Although some comments showed this to be a negative experience, mostly due to the time pressure and extra workload associated with family life or giving online teaching (as documented in Spiro et al., 2021), some people described experiencing this positively as a rest and a chance to work differently on their instrumental practice.

A demographic breakdown appears to support the participants’ comments that having school-aged children and teaching both reduced the amount of practice (55 and 50% reported less practice, respectively). Students, on the other hand, reported more practice than non-students (14% reported more, 14% less, 0% the same and 71% fluctuating practice compared with 7, 53, 13, and 27% in non-students, respectively). The large amount of fluctuating practice in students may reflect the fluctuating motivational levels reported in Philippe et al. (2020). The difference between men and women (18% of men reported practicing less compared to 64% of women) appears to support the finding that women in Switzerland were more likely to reduce their working hours than men (Steinmetz and Monsch, 2021). However, perhaps due to small sample size, none of the above findings were proved to be significant.

### Type of practice

Fifty-five percent of participants spent a larger proportion of practice time on technique compared to their usual, pre-pandemic routine, and this result was found to be significant. This supports the participants’ comments that they used the time to undertake technical work that they would not otherwise have had time to do. The additional comments regarding learning new repertoire, or even learning new instruments reflects the mixed experiences of musicians during the pandemic, and points to moments of productivity and creativity, as documented in Nusseck and Spahn (2021) and Schiavio et al. (2021).

The demographic breakdown showed students spending more time on technique than non-students (71 and 47% reporting more, respectively) and men spending more time on technique than women (73 and 36% reporting more, respectively). These results were not shown to be significant (although the gender effect approaches significance, *p* = 0.065), possibly due to small sample size, but may be linked to the relatively larger amount of practice time reported in these groups above. The majority of those who increased the proportion of technique-practice agreed with the statements that it has had a positive impact on their technique (75% agreed, statistically more likely compared to those who answered “disagree”), and changed how they practice now and will practice in the future (58% agreed). It is notable that the latter statement received less agreement than the former, i.e., some participants acknowledged a positive change in practice that they nonetheless do not plan to incorporate into their future routine. This could be an acknowledgement, in line with the collected statements, that, once concert life resumes, repertoire practice becomes a priority again.

### Online content

Fifty-nine percent of respondents reported “never” watching online content related to technique, such as masterclasses (a statistically significant majority compared to “often”). However, 75% of those who did report an increase in watching this content felt that it had a positive impact upon their own instrumental practice. The proliferation of material on YouTube and other platforms could be considered anecdotal evidence of the production of online content being accelerated by the lockdown periods. Indeed, almost all (95%) respondents in this study engaged in making some kind of audio/video content themselves, in the form of pre-recorded/streamed concerts and tutorials for students. This shows the productivity of a population a majority of whom were out of work or undertaking a reduced amount of work during this period, supporting reports of increased creativity despite the reduction in paid work (Spiro et al., 2021). In the demographic breakdown, teachers were seen to be particularly active in making online tutorials (25% created tutorials only, 31% tutorials and concerts, compared with 0 and 17% for non-teachers, respectively). This is supported by results in a British survey that show instrument teachers being more receptive to use of digital technology than non-teachers, and reporting increased use and a greater willingness to try new technology (Waddell and Williamon, 2019). The comments in our survey and in the literature (Güsewell and Terrien, 2021) show nuanced feedback from teachers that acknowledges both advantages and disadvantages of distanced learning *via* such content.

### Digital tools

Most respondents (68%) reported using digital tools often, and more than in their pre-pandemic practice (54% of respondents). Those who reported increased use of such tools described the experience almost unanimously (and with statistical significance) as improving their technique. However, individual comments point to a more complex relationship, particularly with recording devices, which have elicited both positive and negative feedback. Increased engagement with digital tools was also seen in the respondents purchasing specific software to facilitate new practicing and teaching methods. A particularly frequent use of digital tools was, unsurprisingly, seen in the student population. Notably, there was a statistically significant difference between men and women’s use of digital tools, with 91% of men vs. 45% of women reporting using such tools often. This difference is especially pertinent considering the significant reported improvement associated with such tools. These results seem to support the existence of a digital learning gap between women and men, that has been reported as existing from childhood, e.g., in the context of music education (Colley et al., 1997), and in the context of national and international attempts to unpick the underlying causes of this imbalance (Davaki, 2018). Our results are supported by a survey of digital learning technology in individual settings undertaken on a cohort of 338 amateur, student, and professional musicians in Britain (Waddell and Williamon, 2019). After demographic corrections were made to the data, men reported more usage of audio and video recording, and audio playback devices in musical instrument practice. Moreover, men reported a significantly higher tendency to seek out new technology to aid musical instrument practice, to enjoy learning it, and to enjoy using it. However, although these differences were significant, they were not large. Moreover, there was no significant difference in general (i.e., not music-specific) skills in computer and mobile devise usage, and thus the authors suggest that the established trend of greater digital confidence amongst men and boys may be waning, and the gender gap narrowing, as has been reported elsewhere in the case of music students (Colley and Comber, 2003). However, the above studies were carried out before the COVID-19 pandemic, and it is possible that the pandemic has reversed apparent progress, especially given the reduced working times reported by women during this period (Steinmetz and Monsch, 2021).

### Limitations

The main limitation of this study is the small sample size, particularly in analyzing demographic data, especially when there were multiple choices in response to a specific question. Although we recorded which instrument the respondents played, the sample size (even when grouped by instrumental family) was too small to make related assertions. In addition, this survey was undertaken in autumn 2021; thus, the time that had passed since the pandemic-related concert-free time might have introduced some errors due to inaccurate memory of the participants; e.g., looking more favorably upon this period in retrospect than while it was underway. The self-reporting required in this study may also have introduced some discrepancies, e.g., exaggerating positive or negative responses due to self-conscious behavior during the interview. Since the original call for participation was sent by email, it is possible that the selected respondents were more technologically-aware than the general population.

### Future work

The changes that the period of pandemic-related concert-free time induced in musical practice were in many ways exceptional, and it is expected, or at least hoped, that musicians will not experience such restrictions again. The window for making analyses of this period is closing, as reliability in self-reporting practices during this period reduces with time. However, certain aspects of musical instrument practice that were unique to this period, or trends that were accelerated by it, may provide interesting future case studies under “normal” working conditions, and lead to recommendations for future musical practice. For example, although the minority of respondents reported watching online content, those who did watch it considered it to have had a positive impact upon technique. It may thus be interesting to investigate this effect more closely, and to consider how to broaden the reach of such media, by changing the content, and promoting it differently to musicians. Future work may also involve investigating what aspects of such content musicians find useful, and why, perhaps following up this discussion with an intervention to assess improvements in practice efficiency or performance associated with specific types of content. Similarly, despite the reported perceived benefits of peer-peer learning and engagement (Nielsen et al., 2018), and the efforts made towards online connection during the pandemic (Schiavio et al., 2021), the respondents seemed only rarely to have watched online material produced by their peers. Again, an increased awareness and promotion of such material, may be beneficial to the community. The significant reported improvement in using digital tools appears currently to be benefiting men more than women. Further studies, investment, and promotional work would also be important here to narrow this gap, and to improve these tools further.

## Conclusion

The following main conclusions can be drawn from this study. Firstly, the COVID-19 pandemic-related restrictions significantly changed the amount of practice that musicians undertook, mostly resulting in a reduction or fluctuation in practice habits. Secondly, the pandemic-related concert-free time appears to have had the effect of increasing the proportion of practice time musicians spent on technique. Thirdly, the pandemic did not have a significant effect upon the amount of online content related to technique that musicians watched during the pandemic. However, consuming such content seems to be beneficial to technique. The production of online content, in the form of streamed concerts or tutorials, was prevalent amongst musicians during this time. Finally, the use of digital tools such as metronomes, tuners, and recording devices is widespread amongst musicians. This use increased during the pandemic and was considered beneficial to instrumental technique. Men are more likely to use such tools in their practice than women.

## Data availability statement

The original contributions presented in the study are included in the article/Supplementary material, further inquiries can be directed to the corresponding author.

## Ethics statement

Ethical review and approval was not required for the study on human participants in accordance with the local legislation and institutional requirements. Written informed consent for participation was not required for this study in accordance with the national legislation and the institutional requirements.

## Author contributions

EF conducted the interviews and drafted the first version of the manuscript. PG conducted the statistical analyses and participated in the writing and critical revision of the manuscript. All authors contributed to the article and approved the submitted version.

## Funding

This work was supported by a SPARK grant from the Swiss National Science Foundation to EF (subsidy number 190597).

## Conflict of interest

The authors declare that the research was conducted in the absence of any commercial or financial relationships that could be construed as a potential conflict of interest.

## Publisher’s note

All claims expressed in this article are solely those of the authors and do not necessarily represent those of their affiliated organizations, or those of the publisher, the editors and the reviewers. Any product that may be evaluated in this article, or claim that may be made by its manufacturer, is not guaranteed or endorsed by the publisher.

## Supplementary material

The Supplementary material for this article can be found online at: https://www.frontiersin.org/articles/10.3389/fpsyg.2022.846953/full#supplementary-material

## Footnotes

^1^ https://www.youtube.com/watch?v=3eXT60rbBVk

^2^For a summary of historical COVID19 measures in Switzerland, see: https://www.bag.admin.ch/bag/en/home/krankheiten/ausbrueche-epidemien-pandemien/aktuelle-ausbrueche-epidemien/novel-cov/massnahmen-des-bundes.html#757183649
