# Supplementary material for: An etude for post-pandemic practice: The impact of the COVID-19 pandemic on practice methods and instrumental technique
Source: Front Psychol. 2022 Dec 21;13:846953. doi: 10.3389/fpsyg.2022.846953 (PMC9810800; doi:10.3389/fpsyg.2022.846953)
Supplement: Supplementary file 2 [file Table_2.DOCX]

Supplementary Material

# Tables

Table S1 Summary of demographic data

| Anonymized person code | Gender | Age group 1=20-35, 2=36-50, 3=51-65 | Instrument | Student | School-aged children | Teach |
| --- | --- | --- | --- | --- | --- | --- |
| P1 | m | 1 | Tuba | 1 | 0 | 0 |
| P2 | m | 2 | Trombone | 1 | 1 | 1 |
| P3 | f | 2 | Cello | 0 | 0 | 0 |
| P4 | f | 2 | Flute | 0 | 1 | 1 |
| P5 | f | 2 | Piano | 0 | 0 | 1 |
| P6 | m | 1 | Lute | 1 | 0 | 0 |
| P7 | f | 1 | Cello | 1 | 0 | 0 |
| P8 | m | 2 | Viola | 0 | 1 | 1 |
| P9 | f | 2 | Voice | 0 | 1 | 1 |
| P10 | m | 3 | Trombone | 0 | 1 | 1 |
| P11 | f | 3 | Cello | 0 | 1 | 1 |
| P12 | f | 2 | Shawm | 0 | 0 | 1 |
| P13 | m | 2 | Gamba | 0 | 0 | 1 |
| P14 | m | 1 | Voice | 0 | 0 | 1 |
| P15 | f | 1 | Voice | 0 | 0 | 1 |
| P16 | f | 2 | Bassoon | 0 | 1 | 1 |
| P17 | m | 2 | Piano | 0 | 1 | 1 |
| P18 | f | 1 | Violin | 0 | 1 | 1 |
| P19 | m | 1 | Clarinet | 1 | 1 | 1 |
| P20 | f | 2 | Cello | 0 | 1 | 1 |
| P21 | m | 1 | Percussion | 1 | 0 | 0 |
| P22 | m | 1 | Cello | 1 | 0 | 0 |

Notes: m = male, f = female, 0 = no, 1 = yes

**TABLES with the relative risks**

**Significant effects are written in bold.**

Table S2 Amount of practice compared to usual. Relative risks of answering ‘less’, ‘more’, ‘the same’, ‘fluctuating’ (n=22)

| Contrast | Relative risk | 95% Confidence interval | *p*-value |
| --- | --- | --- | --- |
| Less vs. more | 4.5 | 1.0 – 20.8 | 0.054 |
| Less vs. the same | 4.5 | 1.0 – 20.8 | 0.054 |
| Less vs. fluctuating | 1.0 | 0.4 – 2.5 | 1.0 |
| Fluctuating vs. more | 4.5 | 1.0 – 20.8 | 0.054 |
| Fluctuating vs. the same | 4.5 | 1.0 – 20.8 | 0.054 |
| More vs. the same | 1.0 | 0.1 – 7.1 | 1.0 |
| Change vs. the same | **10.0** | **2.3 – 42.8** | **0.002** |

Notes: “Change” comprises the responses “less”, “more”, and “fluctuating” amount of practice.

Table S3 Proportion of practice time spent on technique compared to usual. Relative risks of answering “larger”, “same”, and “smaller” (n=22)

| Contrast | Relative risk | 95% Confidence interval | *p*-value |
| --- | --- | --- | --- |
| Larger vs. same | 1.5 | 0.6 – 3.7 | 0.37 |
| Larger vs. smaller | **6.0** | **1.3 – 26.8** | **0.019** |
| Same vs. smaller | 4.0 | 0.8 – 18.8 | 0.080 |

Table S4 If you answered larger, agreement with the statement ‘This had a positive impact on my technique’. Relative risks of answering ‘agree’, ‘disagree’, ‘not sure’ (n=12)

| Contrast | Relative risk | 95% Confidence interval | *p*-value |
| --- | --- | --- | --- |
| Agree vs. disagree | **9.0** | **1.1 – 71.0** | **0.037** |
| Agree vs. not sure | 4.5 | 1.0 – 20.8 | 0.054 |
| Not sure vs. disagree | 2.0 | 0.2 – 22.1 | 0.57 |

Table S5 If you answered larger, agreement with the statement: ‘This has changed how I practice now, and will practice in the future’. Relative risks of answering ‘agree’, ‘disagree’, ‘not sure’ (n=12)

| Contrast | Relative risk | 95% Confidence interval | *p*-value |
| --- | --- | --- | --- |
| Agree vs. disagree | 2.3 | 0.6 – 9.0 | 0.22 |
| Agree vs. not sure | 3.5 | 0.7 – 16.8 | 0.12 |
| Not sure vs. disagree | 0.7 | 0.1 – 4.0 | 0.66 |

Table S6 Frequency of watching/reading online content related to technique. Relative risks of answering ‘often’, ‘sometimes’ and ‘never’ (n=22)

| Contrast | Relative risk | 95% Confidence interval | *p*-value |
| --- | --- | --- | --- |
| Never vs. often | **6.5** | **1.5 – 28.8** | **0.014** |
| Never vs. sometimes | 1.9 | 0.7 – 4.7 | 0.19 |
| Sometimes vs. often | 3.5 | 0.7 – 16.8 | 0.12 |

Table S7 Amount of content compared to usual habits. Relative risks of answering ‘more’, ‘less’ and ‘the same’ (n=22)

| Contrast | Relative risk | 95% Confidence interval | *p*-value |
| --- | --- | --- | --- |
| Same vs. more | 1.7 | 0.7 – 4.2 | 0.21 |

Notes: No one answered “less”.

Table S8 If you answered more, agreement with the statement: ‘The majority of this content was produced by my peers’. Relative risks of answering ‘agree’, ‘disagree’, and ‘not sure’ (n=8)

| Contrast | Relative risk | 95% Confidence interval | *p*-value |
| --- | --- | --- | --- |
| Disagree vs. agree | 3.0 | 0.6 – 14.9 | 0.18 |

Notes: No one answered “not sure”.

Table S9 If you answered more, agreement with the statement: ‘watching/reading this content has had a positive impact upon how I practice’ Relative risks of answering ‘agree’, ‘disagree’, and ‘not sure’ (n=8)

| Contrast | Relative risk | 95% Confidence interval | *p*-value |
| --- | --- | --- | --- |
| Agree vs. not sure | 3.0 | 0.6 – 14.9 | 0.18 |

Notes: No one answered “disagree”.

Table S10 Creation of self-made concert streams. Relative risks of answering yes and no (n=22)

| Contrast | Relative risk | 95% Confidence interval | *p*-value |
| --- | --- | --- | --- |
| Yes vs. no | **3.4** | **1.3 – 9.2** | **0.016** |

Table S11 Creation of self-made tutorials. Relative risks of answering yes and no (n=22)

| Contrast | Relative risk | 95% Confidence interval | *p*-value |
| --- | --- | --- | --- |
| Yes vs. no | 0.8 | 0.4 – 1.9 | 0.67 |

Table S12 Use of digital tools during the concert-free time (metronome, tuner, recording, device). Relative risks of answering ‘often’, ‘sometimes’, and ‘never’ (n=22)

| Contrast | Relative risk | 95% Confidence interval | *p*-value |
| --- | --- | --- | --- |
| Often vs. never | **15.0** | **2.0 – 113.6** | **0.009** |
| Often vs. sometimes | 2.5 | 1.0 – 6.4 | 0.058 |
| Sometimes vs. never | 6.0 | 0.7 – 49.8 | 0.097 |

Table S13 Comparison of use of tools with usual practice. Relative risks of answering “more”, “same”, and “less” (n=22)

| Contrast | Relative risk | 95% Confidence interval | *p*-value |
| --- | --- | --- | --- |
| More vs. less | **12.0** | **1.6 – 92.3** | **0.017** |
| More vs. same | 1.3 | 0.6 – 3.2 | 0.51 |
| Same vs. less | **9.0** | **1.1 – 71.0** | **0.037** |

Table S14 If you answered more, agreement with the statement: this use of digital tools have a positive impact on your practice. Relative risks of answering ‘agree’, ‘disagree’, and ‘not sure’ (n=12)

| Contrast | Relative risk | 95% Confidence interval | *p*-value |
| --- | --- | --- | --- |
| Agree vs. not sure & disagree | **11.0** | **1.4 – 85.2** | **0.022** |

*******************************************************************

**Fisher’s exact & Tables for the effects of demographic characteristics on the different outcomes**

Table S15 Amount of practice compared to usual.

|  | Fisher’s exact |
| --- | --- |
| Students vs. non-students | 0.15 |
| Children vs. no children | 0.56 |
| Teach vs. do not teach | 0.31 |
| Female vs. male | 0.13 |

The effects of the demographic characteristics on the amount of practice compared to usual cannot be properly estimated.

Table S16 Proportion of practice time spent on technique compared to usual.

|  | Fisher’s exact |
| --- | --- |
| Students vs. non-students | 0.53 |
| Children vs. no children | 0.41 |
| Teach vs. do not teach | 0.81 |
| Female vs. male | 0.26 |

Tables with the results of the ordered logistic regressions for the four demographic characteristics

Table S16.1 Proportion of practice time spent on technique compared to usual. Students vs. non-students

|  | Odds ratio | 95% Confidence interval | *p*-value |
| --- | --- | --- | --- |
| Students vs. non-students | 0.3 | 0.0 – 2.1 | 0.24 |

Table S16.2 Proportion of practice time spent on technique compared to usual. School-aged children vs. no school-aged children

|  | Odds ratio | 95% Confidence interval | *p*-value |
| --- | --- | --- | --- |
| Children vs. no children | 1.4 | 0.3 – 7.1 | 0.70 |

Table S16.3 Proportion of practice time spent on technique compared to usual. Teach vs. do not teach

|  | Odds ratio | 95% Confidence interval | *p*-value |
| --- | --- | --- | --- |
| Teach vs. do not teach | 1.0 | 0.2 – 5.9 | 1.0 |

Table S16.4 Proportion of practice time spent on technique compared to usual. Male vs. female

|  | Odds ratio | 95% Confidence interval | *p*-value |
| --- | --- | --- | --- |
| Male vs. female | 5.4 | 0.9 – 32.2 | 0.065 |

Table S17 Frequency of watching/reading online content related to technique.

|  | Fisher’s exact |
| --- | --- |
| Students vs. non-students | 1.0 |
| Children vs. no children | 0.21 |
| Teach vs. do not teach | 1.0 |
| Female vs. male | 0.66 |

Tables with the results of the ordered logistic regressions for the four demographic characteristics

Table S17.1 Frequency of watching/reading online content related to technique. Students vs. non-students

|  | Odds ratio | 95% Confidence interval | *p*-value |
| --- | --- | --- | --- |
| Students vs. non-students | 0.8 | 0.1 – 4.7 | 0.80 |

Table S17.2 Frequency of watching/reading online content related to technique. School-aged children vs. no school-aged children

|  | Odds ratio | 95% Confidence interval | *p*-value |
| --- | --- | --- | --- |
| Children vs. no children | 1.0 | 0.2 – 5.5 | 0.97 |

Table S17.3 Frequency of watching/reading online content related to technique. Teach vs. do not teach

|  | Odds ratio | 95% Confidence interval | *p*-value |
| --- | --- | --- | --- |
| Teach vs. do not teach | 0.6 | 0.1 – 3.8 | 0.55 |

Table S17.4 Frequency of watching/reading online content related to technique. Female vs. male

|  | Odds ratio | 95% Confidence interval | *p*-value |
| --- | --- | --- | --- |
| Female vs. male | 1.9 | 0.3 – 10.0 | 0.46 |

Table S18 Amount of content compared to usual habits.

|  | Fisher’s exact |
| --- | --- |
| Students vs. non-students | 1.0 |
| Children vs. no children | 1.0 |
| Teach vs. do not teach | 1.0 |
| Female vs. male | 0.66 |

Tables with the results of the ordered logistic regressions for the four demographic characteristics

Table S18.1 Amount of content compared to usual habits. Students vs. non-students

|  | Odds ratio | 95% Confidence interval | *p*-value |
| --- | --- | --- | --- |
| Students vs. non-students | 0.7 | 0.1 – 4.2 | 0.67 |

Table S18.2 Amount of content compared to usual habits. School-aged children vs. no school-aged children

|  | Odds ratio | 95% Confidence interval | *p*-value |
| --- | --- | --- | --- |
| Children vs. no children | 1.0 | 0.2 – 5.7 | 1.0 |

Table S18.3 Amount of content compared to usual habits. Teach vs. do not teach

|  | Odds ratio | 95% Confidence interval | *p*-value |
| --- | --- | --- | --- |
| Teach vs. do not teach | 0.8 | 0.1 – 6.0 | 0.86 |

Table S18.4 Amount of content compared to usual habits. Female vs. male

|  | Odds ratio | 95% Confidence interval | *p*-value |
| --- | --- | --- | --- |
| Female vs. male | 2.2 | 0.4 – 13.2 | 0.38 |

Table S19 Creation of self-made content concert streams.

|  | Fisher’s exact |
| --- | --- |
| Students vs. non-students | 0.13 |
| Children vs. no children | 1.0 |
| Teach vs. do not teach | 0.27 |
| Female vs. male | 1.0 |

Tables with the results of the logistic regressions for the four demographic characteristics

Table S19.1 Creation of self-made content concert streams. Students vs. non-students

|  | Odds ratio | 95% Confidence interval | *p*-value |
| --- | --- | --- | --- |
| Students vs. non-students | Cannot properly be estimated | | |

Table S19.2 Creation of self-made content concert streams. School-aged children vs. no school-aged children

|  | Odds ratio | 95% Confidence interval | *p*-value |
| --- | --- | --- | --- |
| Children vs. no children | 0.6 | 0.1 – 4.5 | 0.61 |

Table S19.3 Creation of self-made content concert streams. Teach vs. do not teach

|  | Odds ratio | 95% Confidence interval | *p*-value |
| --- | --- | --- | --- |
| Teach vs. do not teach | Cannot properly be estimated | | |

Table S19.4 Creation of self-made content concert streams. Female vs. male

|  | Odds ratio | 95% Confidence interval | *p*-value |
| --- | --- | --- | --- |
| Female vs. male | 0.6 | 0.1 – 4.5 | 0.61 |

Table S20 Creation of self-made content tutorials.

|  | Fisher’s exact |
| --- | --- |
| Students vs. non-students | 0.38 |
| Children vs. no children | 1.0 |
| Teach vs. do not teach | 0.16 |
| Female vs. male | 0.20 |

Tables with the results of the logistic regressions for the four demographic characteristics

Table S20.1 Creation of self-made content tutorials. Students vs. non-students

|  | Odds ratio | 95% Confidence interval | *p*-value |
| --- | --- | --- | --- |
| Students vs. non-students | 0.3 | 0.1 – 2.4 | 0.29 |

Table S20.2 Creation of self-made content tutorials. School-aged children vs. no school-aged children

|  | Odds ratio | 95% Confidence interval | *p*-value |
| --- | --- | --- | --- |
| Children vs. no children | 1.0 | 0.2 – 5.4 | 1.0 |

Table S20.3 Creation of self-made content tutorials. Teach vs. do not teach

|  | Odds ratio | 95% Confidence interval | *p*-value |
| --- | --- | --- | --- |
| Teach vs. do not teach | 6.4 | 0.6 – 68.3 | 0.12 |

Table S20.4 Creation of self-made content tutorials. Female vs. male

|  | Odds ratio | 95% Confidence interval | *p*-value |
| --- | --- | --- | --- |
| Female vs. male | 4.7 | 0.8 – 28.5 | 0.095 |

Table 21 Use of digital tools during the concert-free time (metronome, tuner, recording, device).

|  | Fisher’s exact |
| --- | --- |
| Students vs. non-students | 0.08 |
| Children vs. no children | 0.63 |
| Teach vs. do not teach | 0.16 |
| Female vs. male | 0.06 |

Tables with the results of the ordered logistic regressions for the four demographic characteristics

Table 21.1 Use of digital tools during the concert-free time (metronome, tuner, recording, device). Students vs. non-students

|  | Odds ratio | 95% Confidence interval | *p*-value |
| --- | --- | --- | --- |
| Students vs. non-students | Cannot be properly estimated | | |

Table 21.2 Use of digital tools during the concert-free time (metronome, tuner, recording, device). School-aged children vs. no school-aged children

|  | Odds ratio | 95% Confidence interval | *p*-value |
| --- | --- | --- | --- |
| Children vs. no children | 1.3 | 0.2 – 7.8 | 0.77 |

Table 21.3 Use of digital tools during the concert-free time (metronome, tuner, recording, device). Teach vs. do not teach

|  | Odds ratio | 95% Confidence interval | *p*-value |
| --- | --- | --- | --- |
| Teach vs. do not teach | Cannot be properly estimated | | |

Table 21.4 Use of digital tools during the concert-free time (metronome, tuner, recording, device). Male vs. female

|  | Odds ratio | 95% Confidence interval | *p*-value |
| --- | --- | --- | --- |
| Male vs. female | **12.3** | **1.1 – 131.7** | **0.038** |

Table 22 Comparison of use of tools with usual practice.

|  | Fisher’s exact |
| --- | --- |
| Students vs. non-students | 1.0 |
| Children vs. no children | 0.08 |
| Teach vs. do not teach | 0.75 |
| Female vs. male | 0.39 |

Tables with the results of the ordered logistic regressions for the four demographic characteristics

Table 22.1 Comparison of use of tools with usual practice. Students vs. non-students

|  | Odds ratio | 95% Confidence interval | *p*-value |
| --- | --- | --- | --- |
| Students vs. non-students | 0.8 | 0.1 – 4.5 | 0.78 |

Table 22.2 Comparison of use of tools with usual practice. School-aged children vs. no school-aged children

|  | Odds ratio | 95% Confidence interval | *p*-value |
| --- | --- | --- | --- |
| Children vs. no children | 3.5 | 0.6 – 20.4 | 0.16 |

Table 22.3 Comparison of use of tools with usual practice. Teach vs. do not teach

|  | Odds ratio | 95% Confidence interval | *p*-value |
| --- | --- | --- | --- |
| Teach vs. do not teach | 2.1 | 0.3 – 14.7 | 0.45 |

Table 22.4 Comparison of use of tools with usual practice. Female vs. male

|  | Odds ratio | 95% Confidence interval | *p*-value |
| --- | --- | --- | --- |
| Female vs. male | 0.59 | 0.1 – 3.2 | 0.54 |

**2 Questionnaire script**

(Consent for recording and use of results)

Demographics: Age (20-35, 36-50, 51-65)? Which instrument to you play? Do you teach? Are you a student? Do you have school-aged children?

This questionnaire is about your practicing habits during the COVID-19 pandemic-related concert-free time. This time period includes the strict lockdowns and the periods when concerts were not possible due to the COVID-19 pandemic.

‘Usual’ refers to your pre-pandemic working habits.

This question is about the amount of practice you did during pandemic-related concert-free time.

1. During the period of concert-free time that we experienced due to the COVID-19 pandemic, how did the amount of time you spent practicing compare to usual?

-More

-Less

-The same

- Fluctuating amounts (e.g., you would agree with the statement ‘there were periods when I practiced a lot more than usual, and periods when I practiced a lot less than usual’)

Are there any details you want to add?

This question is about the type of practice that you did in this time.

2. What was the proportion of practice time that you spent on technique compared to usual (examples of ‘technique’ practice: etudes, studies, exercises, scales, repertoire excerpts with a technical goal, general technical work on sound/intonation, etc.)?

- I spent a larger proportion of time on technique than usual
- I spent a smaller proportion of time on technique than usual
- I spent the same proportion of time on technique as usual

Sub-questions:

If you answered larger to the above question, do you agree with the following statements:

This has had a positive impact on my technique

Agree

Disagree

Not sure

This has changed how I practice now, and will practice in the future

Agree

Disagree

Not sure

Any details you want to add?

1. During the pandemic-related concert-free time, did you watch/read online content related to technique (NOT concerts, but: masterclasses, tutorials, podcasts, blogs, articles, etc.)

Often

Sometimes

Never

How does this compare to your usual habits?

More content than usual

Less content than usual

The same amount of content as usual

Sub-questions

If you answered more to the above question, do you agree with the following statement?

Watching/reading this content has had a positive impact upon how I practice.

Agree

Disagree

Not sure

The majority of this content was produced by my peers (musicians around the same age as me, undertaking similar activities to me)

Agree

Disagree

Not sure

Did you take part in making any electronic content

in the form of streamed or pre-recorded concerts?

Yes

No

in the form of video tutorials, masterclasses, or blogs for your colleagues or students?

Yes

No

Any details you want to add?

1. During the pandemic-related concert-free time, did you use digital tools during practice (e.g., metronome, tuner, recording device)

Often

Sometimes

Never

How does this compare to your usual habits?

More than usual

Less than usual

The same as usual

If you answered more to the above question, do you agree with the following statement?

Using these digital tools has had a positive impact upon how I practice.

Agree

Disagree

Not sure

Any details you want to add?

**
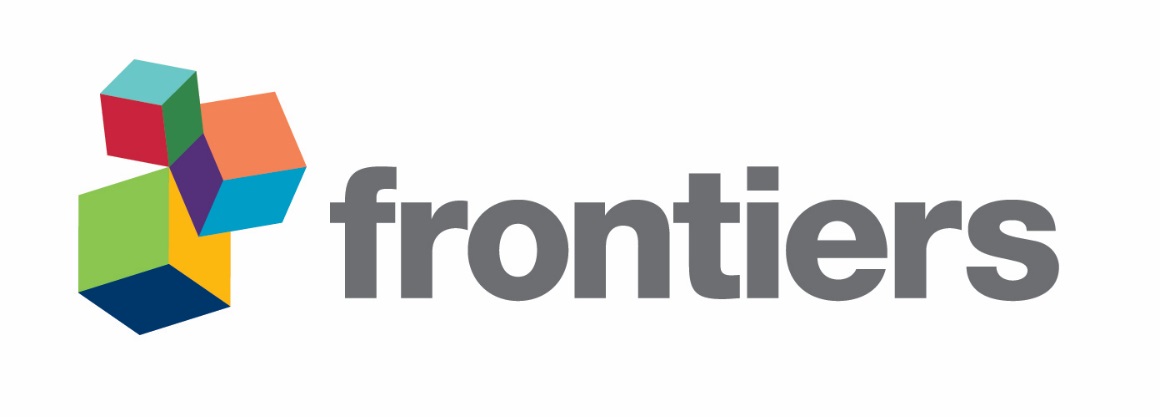
**
